# PROVIDING QUALITY ASSURANCE IN ONLINE GERONTOLOGY COURSES THROUGH A PEER REVIEW NETWORK

**DOI:** 10.1093/geroni/igz038.3545

**Published:** 2019-11-08

**Authors:** Louise M Murray, Enoch Park

**Affiliations:** 1 University of Maryland Baltimore County, Erickson School of Aging Studies, Baltimore County, Maryland, United States; 2 University of North Carolina at Charlotte, Charlotte, North Carolina, United States

## Abstract

In health care and senior housing and care, the challenge of meeting diverse regional workforce needs is increasingly important in higher education. Educators within gerontology are rising to this challenge using multiple platforms, including online education (Carter, Solberg, & Solberg, 2017; Nadash, Miller, Porell, Birchander, Glickman, & Burr, 2014). This poster presents the life cycle of a traditional face to face introductory level graduate gerontology course which was transitioned to a fully online course. This transition was achieved using a structured course development process based on nationally recognized online course quality standards by Quality Matters (QM). Utilizing a team approach, the process incorporated faculty (subject matter experts and course developers), instructional designers, and professional staff in distance education, faculty teaching support, and online learning specialists. QM standards serve to assure quality online instruction, with the goal of providing students with a positive and successful online learning experience. Analysis of this case highlights the role of Certified (QM) peer reviewers who have expertise in Gerontology and can serve as the connector of online gerontology courses offered within programs, between institutions, and globally. Recommendations are presented to improve the pedagogical quality of online courses to attract and retain students in gerontology and aging studies courses. The case for building an initial network of online course peer reviewers to strengthen online teaching and learning within the gerontology profession will be made.

